# The HEALTHGRAIN definition of ‘whole grain’

**DOI:** 10.3402/fnr.v58.22100

**Published:** 2014-02-04

**Authors:** Jan Willem van der Kamp, Kaisa Poutanen, Chris J. Seal, David P. Richardson

**Affiliations:** 1 TNO Food and Nutrition, Zeist, the Netherlands; 2 VTT, Espoo, Finland; 3 Department of Clinical Nutrition, University of Eastern Finland, Kuopio, Finland; 4 Human Nutrition Research Centre, School of Agriculture, Food & Rural Development, Newcastle University, Newcastle upon Tyne, UK; 5 dprnutrition, Croydon, UK

**Keywords:** cereal grains, wholegrain, definition, flour processing

## Abstract

Most cereal products, like white bread, pasta, and biscuits, are based on flour after removal of bran and germ, the two parts of grain kernels containing most of the dietary fibre and other bioactive components. In the past decade, consumers have been rediscovering whole grain-based products and the number of wholegrain products has increased rapidly. In most countries in Europe and worldwide, however, no legally endorsed definition of wholegrain flour and products exists. Current definitions are often incomplete, lacking descriptions of the included grains and the permitted flour manufacturing processes. The consortium of the HEALTHGRAIN EU project (FP6-514008, 2005–2010) identified the need for developing a definition of whole grain with the following scope: 1) more comprehensive than current definitions in most EU countries; 2) one definition for Europe – when possible equal to definitions outside Europe; 3) reflecting current industrial practices for production of flours and consumer products; 4) useful in the context of nutritional guidelines and for labelling purposes. The definition was developed in a range of discussion meetings and consultations and was launched in 2010 at the end of the HEALTHGRAIN project. The grains included are specified: a wide range of cereal grains from the *Poaceae* family, and the pseudo-cereals amaranth, buckwheat, quinoa, and wild rice. The definition also describes manufacturing processes allowed for producing wholegrain flours. This paper compares the HEALTHGRAIN definition with previous definitions, provides more comprehensive explanations than in the definition itself regarding the inclusion of specific grains, and sets out the permitted flour manufacturing processes.

Cereal grain kernels consist of three main parts: endosperm, bran, and germ. In Europe and worldwide most cereal products, like white bread and pasta, are based on kernels or flour after removal of bran and germ, the two outer parts containing most of the dietary fibre and other bioactive components. In population studies, increased intake of whole grain is convincingly shown to be associated with lower risk of cardiovascular disease and type 2 diabetes (1, 2). Results of population studies consistently indicate that average intake of whole grain is far below recommended levels (3–7). However, for the major part these studies are based on old data. More recent data from market research, collected and kept up-to-date by the Whole Grains Council (WGC), show a substantial increase in the past decade of launches of whole grain products: from 2000 to 2010 the number of launches per year has increased from, for example, 300 to more than 3,000 (8). Possibly due to the consistent promotion of wholegrain products, consumption in the United States rose by 23.4% from 2008 to 2010 (9), although the actual average intake of ~14 g/day is still far below the intake of at least 48 g/day recommended in the United States. In Denmark, where wholegrain consumption is actively promoted since 2010, the average intake increased substantially, from 32 g/day in 2000–2004 to 55 g/day in 2011–2012 (10). In Germany, without a wholegrain consumption campaign, a modest increase in the production of wholemeal flour has been observed in recent years (11).

Consumption of wholegrain products is recommended in various ways:

1. Linked to public health – in generic dietary guidelines. For example: Starchy foods such as bread, cereals, rice, pasta, and potatoes are a really important part of a healthy diet. Try to choose wholegrain varieties whenever you can (Food Standards Agency, UK) (12).
2. For specific health benefits, for example, as is stated in the whole grain health claim in the USA: Diets rich in whole grain foods and other plant foods and low in total fat, saturated fat, and cholesterol may reduce the risk of heart disease and some cancers (13).
3. As a preferred way for intake of dietary fibre, as advised in, among others, the Netherlands (14) and Germany (2, 15); for example, at least 30 g of dietary fibre daily, especially from whole-grain products, are recommended (15).

A definition of whole grain is required as a basis for dietary recommendations, labelling – for example, for the list of ingredients on packaged products ‘xx% wholegrain wheat flour’ – and also for nutrition research, for having a clear definition of the materials studied. As has been pointed out, whole grain has been variably defined in different studies so far (16). Defining whole grain as an ingredient is the first challenge, and defining a wholegrain food another. In the first, emphasis should be placed on the way whole grain is currently being used in food processing and thus consumed, as this is the basis of the dietary questionnaires in epidemiological studies and subsequently recommendations for intake.

However, in many countries whole grain and its products are not defined. In a number of countries short definitions exist, stating, as in the Dutch definition: *The word Volkoren (whole grain) can be used for labelling when all components of a cereal grain, as such or after processing, are present in their natural ratio*.

More recently, comprehensive definitions have been developed. A start was made in the United States in 2000 by AACC – with further refinements in subsequent years (17), in 2007 in the United Kingdom (18) and in 2008 in Denmark (19). These definitions include items such as a list of the grains included and specifications of permitted flour manufacturing processes. The AACC International definition (17) is widely cited and used internationally.

In the course of the largest cereal and health-related project ever, the HEALTHGRAIN EU project (FP6-514008, 2005–2010, www.healthgrain.eu), the HEALTHGRAIN consortium identified the need for developing a definition of whole grain with the following scope:

1. It should be more comprehensive than current definitions used in most EU countries.
2. It should be one single definition of whole grain for use across for Europe – where possible equivalent to definitions outside Europe, notably the AACC International definition.
3. It should reflect current industrial flour manufacturing practices.
4. It should be useful in the context of nutrition guidelines and for food labelling purposes.

Such a definition could be used by organisations involved in formulating nutrition guidelines, food inspection agencies, food business operators, risk assessors, and organisations involved in communication about food and nutrition.

The HEALTHGRAIN definition was developed in an interactive and iterative process starting with an enquiry on websites and followed by discussion meetings, formulation of drafts and exchange of views by e-mail. A committee with the late Nils-Georg Asp (Swedish Nutrition Foundation, Sweden), David Richardson (DPR Nutrition Ltd., UK), Kaisa Poutanen (VTT and University of Eastern Finland) and Jan Willem van der Kamp (TNO, the Netherlands) guided the discussions and formulated the definition. Discussions were organised as open meetings, usually in connection with meetings of the HEALTHGRAIN project as shown in Table 1. Attendance ranged from 30 to 50 with a mix of persons from universities and institutes, industries and organisations focusing on communication about food and nutrition. This multidisciplinary group included nutrition scientists, cereal scientists and technologists, plant breeders, flour milling specialists, and experts in regulatory affairs from all over Europe.

**Table 1:** Open meetings from which the whole grain definition was formulated

Venue and dates	Activity
Autumn 2008–March 2009	Enquiry on websites of HEALTHGRAIN and Cereals and Europe^1^
Paris 4-11-2008	Open discussion meeting organised by HEALTHGRAIN
After HEALTHGRAIN Industrial Technology Platform Workshop
Newcastle, UK 23-3-2009	Discussion session • 1st draft definition and e-mail discussion
Part of 3rd Whole Grain Global Summit
La Grande Motte, France 11-6-2009	Discussion Session • 2nd draft definition and e-mail discussion
4th HEALTHGRAIN Annual Meeting
Frankfurt, Germany 17-11-2009	Open discussion meeting organised by HEALTHGRAIN • 3rd draft and finalisation of definition December 2009–February 2010
After HEALTHGRAIN Industrial Technology Platform Workshop
Lund, Sweden 07-05-2010	HEATHGRAIN Whole Grain Definition Official presentation and on-line publication
HEALTHGRAIN Final Conference
**June 2010 – present**	**Presentations in symposia and conferences**
Healthgrain Forum activities^2^ including symposia in conferences	2nd World Congress of Public Health Nutrition, Porto, Portugal 23/25-10-2011
	11th Federation of European Nutrition Societies (FENS) Conference, Madrid, Spain 26/29-10-2011
	4th Whole Grains Global Summit, Minneapolis, USA 20/22-05-2012

1 AACC International Regional Section http://www.cerealsandeurope.net/ (accessed July 2013).

2 http://www.healthgrain.org (accessed July 2013).

In British English, the words ‘whole grain’ and ‘wholemeal’ are used interchangeably when describing flours or foods made from whole grain; the term ‘wholemeal’ is protected in the Flour and Bread Regulations (20). In most other languages, one word is used to describe the inclusion of all components of a grain: Vollkorn (German), Fuldkorn (Danish), and Volkoren (Dutch). In this paper, we generally use ‘whole grain’ also when we are describing products made with wholegrain flour, as is also done in the USA and the UK.

## The HEALTHGRAIN definition of ‘whole grain’

The HEALTHGRAIN definition of whole grain is shown in Table 2.

**Table 2:** The HEALTHGRAIN Consortium definition of whole grain

– Whole grains shall consist of the intact, ground, cracked or flaked kernel after the removal of inedible parts such as the hull and husk. The principal anatomical components – the starchy endosperm, germ and bran – are present in the same relative proportions as they exist in the intact kernel.
– Small losses of components – that is, less than 2% of the grain/10% of the bran – that occur through processing methods consistent with safety and quality are allowed.

This text is part of the full HEALTHGRAIN definition document set out in the Supplemental file (21), with a number of explanations. Key issues, notably the grains included and processing aspects, are further discussed in this paper.

## Grains included

The HEALTHGRAIN consortium decided to develop a definition suitable for both dietary recommendations and labelling purposes. Considering that wholegrain products made from all cereal grains have higher levels of dietary fibre and other bioactive compounds than the equivalent foods made from refined grains and taking into account the benefit of global harmonisation of definitions of whole grain, the same broad range of grains was included as in the AACC International definition (Table 3).

**Table 3:** Grains included in the HEALTHGRAIN whole grain definition

Cereal	Scientific name
Cereals	
Wheat, including spelt, emmer, faro, einkorn, khorasan wheat^1^, durums	*Triticum* spp.
Rice, including brown, black, red, and other coloured rice varieties	*Oryza* spp.
Barley, including hull-less or naked barley but not pearled	*Hordeum* spp.
Maize (corn)	*Zea mays*
Rye	*Secale* spp.
Oats, including hull-less or naked oats	*Avena* spp.
Millets	*Brachiaria* spp.; *Pennisetum* spp.; *Panicum* spp.; *Setaria* spp.; *Paspalum* spp.; *Eleusine* spp.; *Echinochloa* spp.
Sorghum	*Sorghum* spp.
Teff (tef)	*Eragrotis* spp.
Triticale	*Triticale*
Canary seed	Phalaris canariensis*
Job's tears	*Coizlacryma-jobi*
Fonio, black fonio, Asian millet	*Digitaria* spp.
Pseudocereals	
Amaranth	*Amaranthus caudatus*
Buckwheat tartar buckwheat	*Fagopyrum* spp.
Quinoa	*Chenopodium quinoa* Willd. is generally considered to be a single species within the Chenopodioideae
Wild rice**	*Zizania aquatica*

1 Khorasan wheat – also known as Kamut (registered trademark).

* In the first version of the definition document two scientific names were erroneously mentioned: *Phalaris arundinacea and P. canariensis*. The former one is a noxious weed.

** In the first version, wild rice was – incorrectly – listed as a cereal and not as a pseudocereal.

The explanatory text of the HEALTHGRAIN definition states: *This whole grain definition is expected to be useful in the context of nutrition recommendations and guidelines and nutrition claims. Health claims, on the other hand, must be based on documentation of specific effects of grains or grain components in the diet*.

In Europe, the Quantitative Ingredient Declaration (QUID) must include the percentage whole grain in a product in labelling, if the presence of whole grain is emphasised. The HEALTHGRAIN definition provides to this end an overview of the grains that can be included. Under the EC nutrition and health claims regulation (22), the nutrition claims must be on the authorised, positive lists. Although ‘nutrition claims’ make provision for ‘other substances’, the EU list does not include whole grain in this category. So, unless ‘whole grain’ is included as an ‘other substance’ in the authorised list, the HEALTHGRAIN definition can play no role in Europe in the area of nutrition claims.

### Exclusion of oilseeds, legumes, and nuts

As mentioned earlier, the AACC International definition includes the grains listed in Table 3. The US-based WGC launched its official definition of whole grain in 2004 (23). In addition to displaying a list with a wide range of grains of the *Poaceae* family the WGC states: *This list is not meant to be comprehensive, but to include those grains most familiar to consumers. Other cereal grasses from the Poaceae (or Gramineous) family, such as canary seed, Job's tears, Montina, Timothy, Fonio, etc. are also whole grains when consumed with all of their bran, germ, and endosperm*. In line with opinions of AACC International and the FDA, the WGC also states: *Amaranth, quinoa, and buckwheat are not in the Poaceae botanical family, but these ‘pseudo-grains’ are normally included with true cereal grains because their nutritional profiles, preparation, and uses are so similar. Oilseeds and legumes (such as flax, chia, sunflower seeds, soy, chickpeas, etc.) are not considered whole grains by the WGC, the AACC International, or the FDA*.

All further discussions and statements on which grains shall be included are focused on cereal and pseudocereal grains and do not consider the inclusion of any oilseeds and legumes.

### Grains included in definitions related to health claims

In a number of health claims and dietary recommendations, a more restricted number of grains is included. In the early days of legally-based health claims, the USA forged the path for whole grain health claims with the 1999 approval of the first whole grain health claim for both reduced risk of heart disease and cancer (24). This health claim was subsequently revised in 2003 following an industry petition by Kraft (13). A consequence of this modification was the removal of reference to a reduced risk of cancer, so the revised health claim only refers to the reduction in risk of heart disease. This claim includes all cereal grains listed in the AACC International definition. Subsequently, the UK JHCI published its generic health claim in 2002 (25), followed in 2003 by the Swedish Code whole grain health claim (26). The UK claim included ‘major cereal grains such as wheat, rice, maize, and oats’ and the Swedish claim just wheat, rye, oats, and barley. This trend for allowing a more limited number of grains is also observed in the general discussions on health claims in the past decade, tending towards requiring for health claims convincing/conclusive evidence of health benefits instead of the probable level of evidence that is usually considered to be sufficient for dietary recommendations. Frølich and Åman (26) made a strong plea for listing in a definition of whole grain-only cereal grains where health benefits are substantiated both by epidemiology and intervention studies, and Frølich also pointed out differences between different whole grains (27). Frølich and Åman proposed to start with a limited number of grains in the definition – their paper implicitly suggests wheat, rye, oats, and barley – and to extend the list only when more knowledge becomes available.

The trend towards requiring convincing/conclusive evidence culminated in the evaluation by the European Food Safety Agency (EFSA) of health claims submitted after publication of Regulation 1924/2006 on nutrition and health claims made on foods (22). This Regulation mentions a food category, a food or one of its constituents as eligible for a health claim. However, the EFSA opinions rejected the whole grain (and fibre) claims as a food category and decided only to allow health benefits relating to specific grains. Furthermore, the original health claims in the UK and Sweden were based on epidemiological evidence alone, but EFSA now requires conclusive evidence from human intervention studies and is applying a pharmaceutical approach to assess the totality of the available data (28). To date, only a number of well-characterised fibres and brans from specific cereal grains – wheat, rye, barley, and oats–have obtained a positive EFSA opinion and are included in the positive list of authorised claims (29). As the EFSA opinions and now the Regulation supersede all the previous claim activity, all earlier claims in Europe, such as those in the UK and Sweden have now been withdrawn (30).

### Grains included in definitions related to dietary recommendations and labelling

Compared with health claims, a larger number of grains have been included in documents providing guidance to labelling and in dietary recommendations

1. the UK Institute of Grocery Distribution (IGD) Whole grain guidance note (18), aiming at providing guidance to supermarkets and related retailers, includes cereals and generally accepted pseudocereals, as is done in the AACC definition.
2. in Sweden, the Scandinavian Keyhole system for healthy eating (26) lists the eight cereal grains most consumed in Sweden: wheat, rye, oats, barley, maize, rice, sorghum, and millet, but does not include pseudocereals.
3. In Denmark the same applies as in Sweden, with the addition of triticale (19).

An overview of the grains included and the purpose of the definition in the documents mentioned above and in earlier paragraphs is presented in Table 4. In this table, labelling and dietary recommendations are placed in one column, since the related documents do not clearly differentiate between these purposes.

**Table 4:** Grains included in whole grain definitions related to purpose

Whole grain definitions		Purpose of definition	
	
Issuing body	Grains included	Labelling, dietary recommendations	Health claims
AACC International/FDA	‘All’ cereals and pseudocereals	Yes	Yes ≥51% whole grain >5% fibre
UK Joint Health Claim Initiative, 2002	Major cereal grains, such as wheat, rice, maize, and oats	No	Yes ≥51% whole grain
UK IGD Whole grain guidance note, 2007	Cereals and generally accepted pseudocereals	Yes	No
Sweden, Health claim code of practice, 2003	Wheat, rye, oats, barley	No	Yes ≥50% whole grain
Scandinavian Keyhole for healthy eating, 2010	Wheat, rye, oats, barley, maize, rice, millet, sorghum	Yes	No
HEALTHGRAIN (2010)	‘All’ cereals and pseudocereals	Yes	No

## Discussion

As indicated above, dietary recommendations range from generic and qualitative to those that are more specific and quantitative. A widely used generic dietary recommendation is ‘eat more fruits and vegetables’. This is comparable to ‘let your grains be whole’. Such recommendations reflect evidence from observational studies and widely supported health messages from a generic, public health perspective. These generic dietary recommendations do not require the health benefits of specific fruits, vegetables or whole grains to be based on evidence from human intervention studies, but rather on the totality of the available data from all sources of evidence, such as meta-analyses (1, 2, 31) Wholegrain products are recommended as a preferred way for securing a sufficient intake of dietary fibre in a number of more specific recommendations. For such recommendations a list of whole grains may be provided focusing on grains with higher levels of dietary fibre. An even more restricted list may apply for health claims, when conclusive evidence is required from human intervention studies. However, the use of lists with a limited number of cereal grains for specific purposes does not detract from the need for having, as a start, a definition for the food category, as adopted by HEALTHGRAIN, and preceded by AACC International, the WGC and the UK IGD, including all cereal grains of the *Poaceae* family plus well known pseudocereals.

## Processing

In commonly used milling processes grain kernels are crushed and divided over various milling streams. These streams are fractions transported separately in the flourmill and recombined in a later stage for the production of wholemeal flour. Before crushing, the very outer layer may be removed by peeling. The HEALTHGRAIN definition includes recombination of milling streams – refined flour, endosperm, and bran – both in the flour mill and by the producer of consumer products, as will be discussed in detail below, as well as the option of removal of some of the outer part of grain kernels: *Small losses of components* – *that is, less than 2% of the grain/10% of the bran* – *that occur through processing methods consistent with safety and quality are allowed*. This option is included because mycotoxins, agrochemicals, and microbial contaminants tend to be concentrated in the very outer pericarp layer (32). This 2% removal is also included in the definition in Switzerland (33). In Germany removal is mentioned in the definition, but without an exact percentage (34). Since this removal of the outer layers is widely applied by millers and since the HEALTHGRAIN definition aims at reflecting current production processes, we decided to add this option. In the explanatory text added in the definition document the following remark is made about recombination: *Recombination per grain and per variety will result in some fluctuations in the ratios of endosperm, bran, and germ between batches of flour and products. There should, however, be no significant nutritional losses, and differences should be no greater than normally found from season to season or between varieties*.

Over 90% of the wholegrain flours of wheat, rye, barley, oats, and maize in food supply chains is created through milling processes where kernels are broken, separated into milling streams containing endosperm rich (white) flour, germ, and various bran fractions, and then combined in fixed ratios. For flour with a long shelf life, enzymes promoting rancidity in the germ are inactivated by heat treatment of the germ fraction (35); for the same reason rice bran can be stabilised. These ‘modern milling’ processes ensure a longer shelf-life and constant flour quality in terms of composition and processability compared with the traditional stone-grinding processes, where grain is usually milled without separation and recombination. In stone grinding, all components usually are included in the flour, but partial removal of the bran and germ is also an option.

Recombination may take place both at the flour mill and during production of the final product. With modern milling processes the recombined flour may be composed of white flour, germ, and bran originating from different batches of the grain. Occasionally the question is raised whether recombination should be allowed for wholegrain products, since the final composition may not be exactly the same as that of wholegrain flour produced by stone-grinding processing. The following arguments were put forward in the in-depth discussions on this issue when the HEALTHGRAIN definition was drafted:

1. Modern milling processes and heat treatment of the germ may affect the composition of the flour in different ways compared with stone grinding. Generally speaking, intact grains are not consumed as such – they have to be processed before they can be included in the diet. Any milling process will have a greater or less impact on the composition of the flour. Dry processing, such as milling, has considerably less impact on the composition than processing in wet systems, such as dough fermentation, baking, and extrusion, which are not reviewed here. An overview of impact on nutrition and health benefits of a wide range of dry and wet processing operations and of variations in product structure is presented in (27). A good illustration on the possible impact of wet processing is the formation of acrylamide, due to heat treatment, for example during baking, extrusion or in other ways of systems containing flour, sugars, and water. Wet processing can also result in beneficial nutritional effects, such as formation of resistant starch during baking, or the release of bioactive components during sourdough fermentation (36, 37).
2. The composition of wholegrain flour of one type of grain shows major variations. Studies before HEALTHGRAIN, and in HEALTHGRAIN itself with a focus on wheat, have shown that the composition of whole grain is affected both by genetic (cultivar) and environmental (agronomic) factors. In the HEALTHGRAIN studies, cultivars could be differentiated according to high and low levels of fibre and other bioactive substances: 1) high fibre, high bioactives; 2) high fibre, low bioactives; 3) low fibre, high bioactives; and 4) low fibre, low bioactives (38). Quality control and good management practices help ensure that wholegrain flours made by the recombination of fixed ratios of milling streams have a consistent composition at least as good as those observed in flours made by stone grinding of individual batches and cultivars.
3. The great majority of the research supporting the health benefits of whole grains has been based on the consumption of foods made with flours prepared from recombined whole grain components. This holds for all the published observational studies and also for most human intervention studies, since recombination is/has been the method used to produce flours used in the manufacture of (almost) all commonly consumed wholegrain products.
4. Any process will offer opportunities for fraudulent or incompetent producers to operate incorrect procedures. One may, for example, add back a lower amount of bran – aiming at a better performance in baking – or only part of the germ, since germ as such can have a high economic value. Therefore, the HEALTHGRAIN definition has included the condition that production of wholegrain flour and wholegrain-based products must follow appropriate Good Manufacturing quality systems.

## Use of the definition

With the HEALTHGRAIN definition, guidance is provided to all those involved in grain production chains. These include flour millers and other grain processors, producers of final products, regulators and food inspection agencies, non-governmental organisations, such as WHO, Codex Alimentarius, and public health organisations focusing on heart health, diabetes and cancer, researchers and consumers and agencies communicating to consumers. The definition can serve as a basis for listing the composition – for example, stating ‘with xx% wholegrain (wheat) flour’. The definition is similar to the AACC International definition as used in the USA, but specifies more explicitly processing issues. Unlike the AACC International definition, the HEALTHGRAIN definition aims only to provide a basis for labelling and nutrition guidelines and recommendations, and not for health claims. The issue as to when a product can be named a wholegrain product is not covered. Here, widely diverging practices and regulations exist in Europe and world wide – and no regulations at all exist in many cases. It is hoped that the HEALTHGRAIN definition will assist in production and labelling of foods rich in whole grain, and that consumers will gradually become more conscious about the type and quality of cereal ingredients in foods in relation to health and wellbeing.
